# Behaviour of iPSC-Derived and Donor-Derived Hepatic Stellate Cells in Organoid Cultures Is Highly Variable and Contributes to Fibrotic Outcome

**DOI:** 10.3390/cells15171556

**Published:** 2026-08-28

**Authors:** Yuwei Yang, Mina Kazemzadeh Dastjerd, Ayla Smout, Vincent Merens, Burak Toprakhisar, Catherine M. Verfaillie, Christophe Chesné, Leo A. van Grunsven

**Affiliations:** 1 Liver Cell Biology Research Group, Faculty of Medicine and Pharmacy, Vrije Universiteit Brussel, 1090 Brussel, Belgium; yuwei.yang@vub.be (Y.Y.); minakaz@kth.se (M.K.D.); ayla.smout@vub.be (A.S.); vincent.merens@medisin.uio.no (V.M.); burak.toprakhisar@vub.be (B.T.); 2 Stem Cell Institute Leuven, Katholieke Universiteit Leuven, 3000 Leuven, Belgium; catherine.verfaillie@kuleuven.be; 3 Biopredic International SARL, Parc de la Bretech, 35760 Saint Grégoire, France; c.chesne@wepredic.com

**Keywords:** iPSC, single-cell RNA sequencing, liver fibrosis, hepatic stellate cell activation, liver organoid

## Abstract

Hepatic stellate cells (HSCs) are central drivers of liver fibrosis, yet their heterogeneity is often overlooked in human liver organoid models. Here, we investigated how HSC-intrinsic variability influences fibrotic phenotypes in multicellular liver organoids using induced pluripotent stem cell-derived HSCs (iHSCs) and primary HSCs (pHSCs). iHSCs derived from two different iPSC lines exhibited distinct differentiation phenotypes towards iHSCs. Consistently, co-culture of the hepatocyte-like cell line HepaRG with these two iHSC populations generated organoids with distinct fibrotic and hepatic features. BJ1-iHSC/HepaRG organoids contained an approximately 2.9-fold higher proportion of CYP3A4-positive cells, whereas Sigma-iHSC/HepaRG organoids showed around a 4-fold higher percentage of Sirius red-positive area. Single-cell RNA analysis further showed that the two iHSC populations differed in cell cycle distribution, HSC-related signatures, extracellular matrix-associated pathways, and matrisome enrichment. Similarly, human pHSCs cultured as organoids with HepaRG showed marked donor-dependent differences at baseline and after single acetaminophen, repeated thioacetamide or transforming growth factor beta treatment. Together, our findings suggest that HSC heterogeneity, whether iPSC-derived or donor-derived, is an important factor in fibrotic output and response variability in human liver organoid models and should be considered in model design, interpretation and comparison between studies.

## 1. Introduction

Liver fibrosis is a common pathological consequence of chronic liver injury. Hepatic stellate cells (HSCs) play a central role in fibrogenesis [1]. Upon injury, HSCs become activated and acquire a myofibroblast-like phenotype. The activated HSCs increase the deposition of extracellular matrix (ECM), primarily collagen type I [2], and interact with other cells resident in or infiltrated into the liver [3,4,5,6]. Although animal models and conventional 2D culture systems focusing on HSCs have provided important insights into fibrogenic mechanisms, there is still a gap between these models and the complexity of human liver biology, and they often show limited translational relevance [7]. In recent years, human liver organoids have emerged as promising platforms to model liver injury and fibrosis in a more physiologically relevant context. In such systems, hepatocyte-like cells such as HepaRGs [8] are commonly incorporated to provide a functional parenchymal component, while HSCs are required to recapitulate stromal activation and ECM remodelling.

Induced pluripotent stem cell-derived HSC-like cells (iHSCs) have emerged as an attractive and renewable cell source for human liver fibrosis modelling. Previous studies [9,10] have largely focused on differences between differentiation strategies and culture protocols, and these methodological effects on iHSC phenotype have been increasingly recognized. In contrast, variation between iPSC sources is often treated mainly as evidence of protocol reproducibility, with successful differentiation across multiple lines interpreted as support for method robustness. As a result, potential line-dependent differences in the resulting iHSC populations are frequently underappreciated [9,11]. Whether such source-dependent variation is sufficient to alter fibrosis-related and hepatic phenotypes in multicellular liver organoid models remains insufficiently explored. Similarly, this may also apply to primary human HSC-based models, where commercial batches and cell sources are often assumed to be broadly comparable despite limited direct evaluation of their functional equivalence [12,13,14].

In this study, we investigated how HSC source-dependent heterogeneity influences fibrotic phenotypes in 2D culture and human liver organoid models. We first compared HSCs derived from two iPSC lines and found that they exhibit distinct fibrogenic marker patterns during 2D differentiation and when co-cultured in 3D with HepaRG cells. To further define the basis of these differences, we performed single-cell RNA analysis and identified distinct transcriptional states associated with differences in proliferative activity, activation-associated pathways, and ECM-related programmes. Finally, we extended our analysis to primary HSCs (pHSCs) from different donors and showed that a similar variability also affects pHSC-based organoid models. Together, our findings indicate HSC source-dependent heterogeneity is an important contributor to response variability and matrix-producing behaviour in human liver fibrosis models examined here.

## 2. Materials and Methods

A schematic overview of the main cell sources, culture systems and experimental readouts used in this study is provided in Supplementary Scheme S1.

### 2.1. iPSC Culture and Maintenance

The human iPSCs Sigma (IPSC0028-1VL; Sigma-Aldrich, St. Louis, MO, USA) and BJ1 derived from human foreskin fibroblasts (ATCC CRL-252228; RRID: CVCL_3653) lines were cultured on Laminin 521 (LN521-05; BioLamina, Sundbyberg, Sweden) coated plates and maintained in Essential 8 Flex medium (A2858501; Thermo Fisher Scientific, Waltham, MA, USA). Cultured iPSCs were detached with StemPro Accutase (Thermo Fisher Scientific, A11105-01) to obtain a single-cell suspension and then seeded on Laminin-coated plates in Essential 8 Flex medium supplemented with 1:100 diluted RevitaCell (A2644501; Thermo Fisher Scientific). All research performed using iPSCs was approved by the Human Ethics Committee at UZ Brussel, Brussels, Belgium (B.U.N. 1432021000423).

### 2.2. Differentiation of iPSCs Towards Hepatic Stellate Cells (iHSCs)

iPSCs were differentiated into iHSCs based on previously published protocols [15,16,17]. Briefly, iPSCs were dissociated into a single-cell suspension by using StemPro Accutase and seeded on Laminin-coated plates at 25,000 cells/cm^2^ in mTeSR™1 medium (85850; Stemcell Technologies, Vancouver, BC, Canada) with 1:100 diluted RevitaCell. The iHSC differentiation started by changing the medium to liver differentiation medium (LDM) [18] when the cells reached a confluency of 60–70%. The following growth factors were added into LDM to induce differentiation stages, and medium was renewal every 2 days: on day 0, 50 ng/mL of BMP4 (120-05; PeproTech, Cranbury, NJ, USA) was supplemented until day 4; on day 4, 50 ng/mL of BMP4 and 20 ng/mL of aFGF (PeproTech, 100-17A) were added; on day 6, 20 ng/mL of aFGF, 100 µM of palmitic acid (Sigma-Aldrich, P5585) and 5 µM of retinol (R7632; Merck, Darmstadt, Germany) were added; on day 8, 20 ng/mL of aFGF was removed, and 100 µM of palmitic acid and 5 µM of retinol were added until day 10. On day 10, the cells were passaged 1/3 using 0.05% Trypsin-EDTA (25300-054; Invitrogen, Waltham, MA, USA). Cells were incubated at 37 °C, scraped from plates and dissociated by gently pipetting. The cells were subsequently maintained in high-glucose Dulbecco’s modified Eagle’s medium (DMEM) (DMEM-HPA; Capricorn, Ebsdorfergrund, Germany) with 2.5% HEPES Buffer solution (Capricorn, HEP-B), 1% Penicillin-Streptomycin (15140122; Gibco, Waltham, MA, USA) and 10% Fetal Bovine Serum (FBS), supplemented with RevitaCell. The Passage 1 iHSCs (iHSC P1) were dissociated again when they reached 90% confluency, and the cells were frozen in DMEM with 10% DMSO and 20% FBS at approximately 2 million cells/mL for further use. The frozen cells were thawed and seeded in one 6-well plate for liver organoid culture.

### 2.3. Primary HSCs

Post-operative human liver pieces were kept temporarily in IGL-1 solution, a commercial organ preservation solution from Institut Georges Lopez (IGL group, IGU.IGL-1 REV07), for no longer than 2 h before cell isolation using the method of Cools et al. [17]. Briefly, the human liver pieces were perfused with perfusion buffer 1, followed by perfusion buffer 2 containing 0.5 mg/mL of Pronase E (Merck, 1074330005). A subsequent 5 min perfusion was performed with perfusion buffer 2 containing 0.25 mg/mL Collagenase P (11213873001; Roche, Basel, Switzerland), using the buffer compositions reported by Cools et al. [17]. The liver tissues were then mechanically minced and digested for 20 min at 37 °C in perfusion buffer 2 containing 0.5 mg/mL Pronase E and 0.5 mg/mL Collagenase P. After 5 and 10 min of digestion, one drop of sodium hydroxide (pH 14) was added to the beaker. The resulting cell suspension was then filtered and centrifuged for 7 min at 50 g to pellet hepatocytes. The supernatant was subsequently centrifuged for 8 min at 1750 RPM, and the remaining cell pellet was incubated for 3 min at room temperature with red blood cell lysis solution. After washing with perfusion buffer 2, the cell pellet was resuspended in an 18% Nycodenz solution (1002424, Axis Shield Diagnostics, Dundee, UK) with DNase I (Roche, 10104159001). This suspension was consecutively topped off with 12% and 8.2% Nycodenz solutions, and centrifuged at 1450 g for 20 min. The HSCs located between the Nycodenz gradients were collected, resuspended in PBS, and centrifuged again at 1750 RPM for 8 min. The final cell pellet was resuspended and seeded in DMEM supplemented with 20% FBS, which was replaced by DMEM containing 10% FBS from the following day onwards. The primary HSCs used for the BP organoids were from Biopredic and cultured in DMEM 10% FBS. Primary HSCs were used until Passage 4–5. Available donor and related information for the primary HSCs, including gender, age, liver disease, medication, cell number available for culture, and viability where available, is summarized in Table 1.

### 2.4. Liver Organoid Cultures

The cryopreserved differentiated HepaRGs (Biopredic, Saint-Grégoire, France) were recovered in HTM thaw/seed medium (Williams E medium, supplemented with 1% Glutamax and ADD670 supplement from Biopredic). The HSCs were released from the culture plates with 0.05% Trypsin-EDTA and resuspended in HTM medium. The viability of cells was determined with trypan blue staining. The organoid seeding procedure was described previously [19,20]. Briefly, HSCs and HepaRG were seeded in 96-well ultra-low attachment U-bottom plates at a ratio of 2:1 in 10 µL of HTM medium per well. 24 h after seeding, 100 µL of HC0DM culture medium (Williams E medium, 1% Glutamax supplement, and ADD610 supplement from Biopredic) was added to each well. Before switching to HFM maintenance medium on day 4 of the 3D culture, the medium was replaced once more with HC0DM (Williams E medium, 1% Ultraglutamine, ADD650 supplement (Biopredic), 0.5% DMSO, 10 ng/mL of HGF (PeproTech, 100-39), and 2 ng/mL of EGF (PeproTech, 315-09)). Thereafter, every other day, 100 µL of old medium was replaced with 100 µL of fresh HFM.

### 2.5. Cell Viability Assay

Paracetamol (APAP) (Merck, A7085) was dissolved in organoid culture medium. After 72 h of treatment, the ATP assay was performed using the CellTiter-Glo Luminescent Cell Viability Assay (Promega, G7571), according to the supplier’s instructions. The luminescence signal was measured by a GloMax (Promega, GM3000). Raw luminescence values were normalized to the mean luminescence of the corresponding control group within each independent experiment or batch and expressed as ATP levels relative to control (%).

### 2.6. RNA Extraction and RT-qPCR

RNA was extracted from cells using the PureLink RNA Mini Kit (Thermo Fisher Scientific). For spheroid samples, six spheroids were pooled for each condition. Reverse transcription was performed using M-MLV Reverse Transcriptase with random primers according to the manufacturer’s instructions (Promega, M1701). qPCR reactions were run on a QuantStudio 3 Real-Time PCR System (Applied Biosystems, Foster City, CA, USA) with GoTaq qPCR Master Mix containing BRYT Green Dye according to the manufacturer’s protocol. All primers used in this manuscript are listed in Table 2.

### 2.7. Sample Preparation for Split Pool Ligation-Based Transcriptome Sequencing (SPLiT-seq)

0.1 to 4 million cells were collected in pre-chilled Falcon tubes coated with 1% Gibco BSA Fraction V and centrifuged for 8 min at 1750 RPM. The collected cell pellet was resuspended in 375 µL of Cold Prefixation Buffer (Parse Bioscience, Seattle, WA, USA) with 25 µL of 7.5% BSA Fraction V and filtered through a 40 µm strainer. 125 µL of Cell Fixation Solution (Parse Bioscience) was added to the cell suspension and mixed well by pipetting up and down. After incubation on ice for 10 min, 40 µL of Cell Permeabilization Solution (Parse Bioscience) was added to the tube and mixed well by pipetting. After incubation on ice for 3 min, 500 µL of Cell Neutralization Buffer (Parse Bioscience) was added and mixed by pipetting. The cells were then centrifuged for 8 min at 1750 RPM, and the pellet was resuspended in 75 µL of cold Cell Buffer (Parse Bioscience) and returned to ice. The cells were filtered again through a 40 µm strainer into a new 1.5 mL LoBind Eppendorf and stored on ice. Next, 1.25 µL of DMSO was added, mixed well and incubated on ice for 1 min. This step was repeated twice for a total addition of 3.75 µL of DMSO and mixed gently, avoiding making bubbles. The cells were counted, and the tubes were stored in a freeze box at −80 °C. Further barcoding, amplification, and library preparation for sequencing were done by following the user manual of the Evercode WT Mini v2 kit provided by Parse Bioscience. The sequencing was performed on an Illumina NovaSeq 6000 (Brightcore VUB).

### 2.8. RNA Sequencing Analysis

Trailmaker^TM^ (Parse Biosciences) was used to analyze the single-cell RNA-sequencing data. The raw FASTQ files were processed using the split-pipe v1.3.1 (Parse Biosciences). Read quality was assessed using FastQC version 0.12.1-Java-11. The reads were aligned to the human hg38 reference genome built with Homo_sapiens. GRCh38.109 using STAR version 2.7.11a-GCC-12.3.0 [21]. SAM/BAM processing was performed using SAMtools version 1.18-GCC-12.3.0. Cell barcode processing, molecule counting, cell calling, and generation of digital gene expression matrices and cell metadata were performed within split-pipe v1.3.1. Downstream analysis was performed using Seurat version 5.0.1 [22]. The doublets were predicted and removed by the package scDblFinder version 1.16.0 [23]. The plots in the figures were drawn by ggplot2 version 3.4.4. The GSEA and enriched GO analysis were performed using clusterProfiler version 4.10.0 [24,25,26] and ssGSEA version 2.0 [27]. The similarity scores of gene sets were calculated by the Seurat function AddModuleScore or the AUCell package version 1.24.0 [28].

### 2.9. Immunofluorescence Staining and Analysis

Cells were fixed in 4% formaldehyde solution (Merck, 1004968350). For staining of 2D cultured cells, cells were washed in PBS containing 0.1% Triton X-100 (Sigma-Aldrich) and then incubated in pre-diluted primary antibody incubating solution (PBS-based 0.1% Triton X-100, 1% BSA) for at least 2 h at room temperature. Primary antibodies were removed by washing 5 times with 0.1% Triton X-100 PBS, and cells were incubated in secondary antibody incubating solution (PBS-based 0.1% Triton X-100, 1% BSA) for 1 h at room temperature. Following 5 PBS washes, cells were imaged by EVOS M7000 (Thermo Fisher Scientific). For immunofluorescence staining of liver organoid sections, 5 µm paraffin sections were incubated at 60 °C for 30 min, followed by deparaffinization and rehydration. Antigen retrieval was performed in the Dako antigen retrieval solution (S169984-2; Dako, Glostrup, Denmark) at 95 °C for 20 min. Slides were then allowed to cool to room temperature. Sections were permeabilized with PBS containing 0.1% Tween-20 and blocked with 2% BSA. Primary and secondary antibodies were applied sequentially and diluted in 1% BSA, with washing steps performed between antibody incubations. All antibodies and relative dilution rates used for staining are given in Table 3. The cell number and positive area were counted or measured in Fiji version Java 1.8.0_322 [29] and QuPath version 0.7.0 [30].

### 2.10. Statistics

Statistical tests were carried out using GraphPad Prism 10. For datasets with more than three samples (*n* > 3), normality was assessed using the Shapiro–Wilk test. To compare more than 3 groups, unpaired one-way ANOVA was used. Comparisons between 2 groups were analyzed using an unpaired *t*-test. Other statistical details can be found in the figures’ legend. Statistical significance is indicated by exact *p* values or as asterisks (* *p* < 0.05, ** *p* < 0.01, *** *p* < 0.001).

## 3. Results

### 3.1. Distinct Fibrogenic Phenotypes Emerge During Differentiation of BJ1- and Sigma-Derived iHSCs

**Figure 1:**
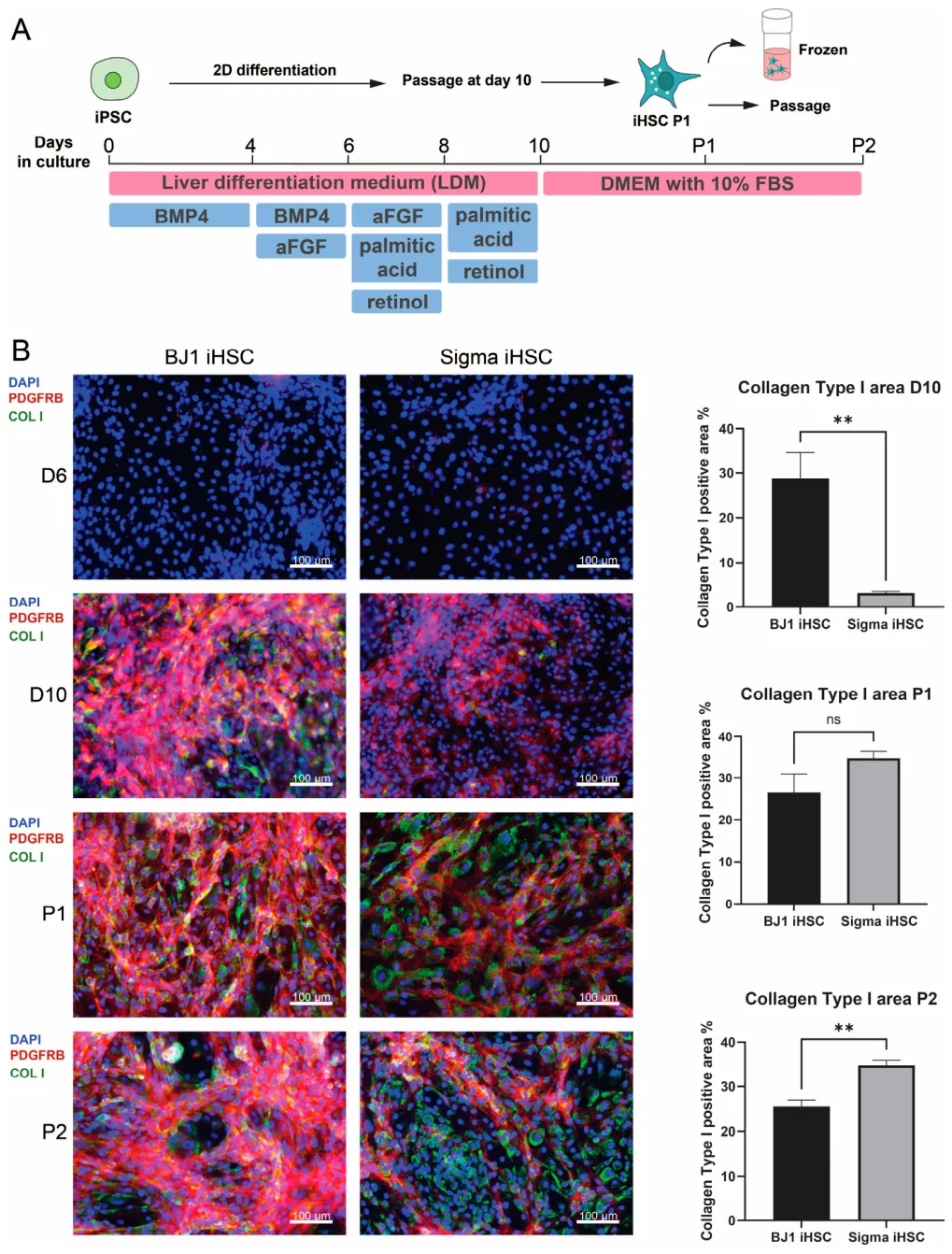
Distinct fibrogenic features emerge during differentiation of BJ1- and Sigma-iPSCs towards iHSCs. (**A**) Schematic overview of the differentiation workflow from iPSCs to iHSCs. (**B**) Representative immunofluorescence images of BJ1-iHSCs and Sigma-iHSCs at differentiation day 6 (D6), day 10 (D10), Passage 1 (P1) and Passage 2 (P2), stained for DAPI, PDGFRB, and collagen type I. Quantification of the percentage of collagen type I-positive area at D10, P1 and P2 is shown in the right panel. Data are presented as mean ± SEM. ** *p* < 0.01, Scale bar: 100 μm. *n* = 3–4 independently differentiated wells per condition, with one image quantified per well.

As iPSC-derived HSCs are increasingly used as a renewable stromal component in multicellular liver models, we first asked whether iHSCs generated from different iPSC sources could already display divergent phenotypes during 2D differentiation. To address this, we compared BJ1-iHSCs and Sigma-iHSCs at matched differentiation stages following a previously published protocol [16,17] (Figure 1A). Immunofluorescence staining for platelet-derived growth factor receptor beta (PDGFRB) and collagen type I at day 6 (D6), day 10 (D10), Passage 1 (P1) and Passage 2 (P2) revealed clear stage-dependent differences between the two populations (Figure 1B). At D6, both BJ1-iHSCs and Sigma-iHSCs showed barely detectable levels of PDGFRB or collagen type I, consistent with an incompletely differentiated state [18]. By D10, however, BJ1-iHSCs displayed strong expression of both PDGFRB and collagen type I, whereas Sigma-iHSCs showed comparatively weaker staining. At later passages, collagen type I expression further changed in a passage-dependent manner: no significant difference in collagen type I-positive area was detected between BJ1-iHSCs and Sigma-iHSCs at P1, whereas at P2, BJ1-iHSCs showed a significantly lower collagen type I-positive area compared with Sigma-iHSCs. Together, these findings indicate that BJ1-iHSCs and Sigma-iHSCs respond differently to similar differentiation cues. Given that these differences were already evident in 2D cultures, we next asked whether iHSCs from different iPSC sources would also differentially influence fibrosis-related and hepatic phenotypes in multicellular liver organoids.

### 3.2. HSCs from Different Sources Influence Fibrosis and Hepatic Functionality in Liver Organoids

**Figure 2:**
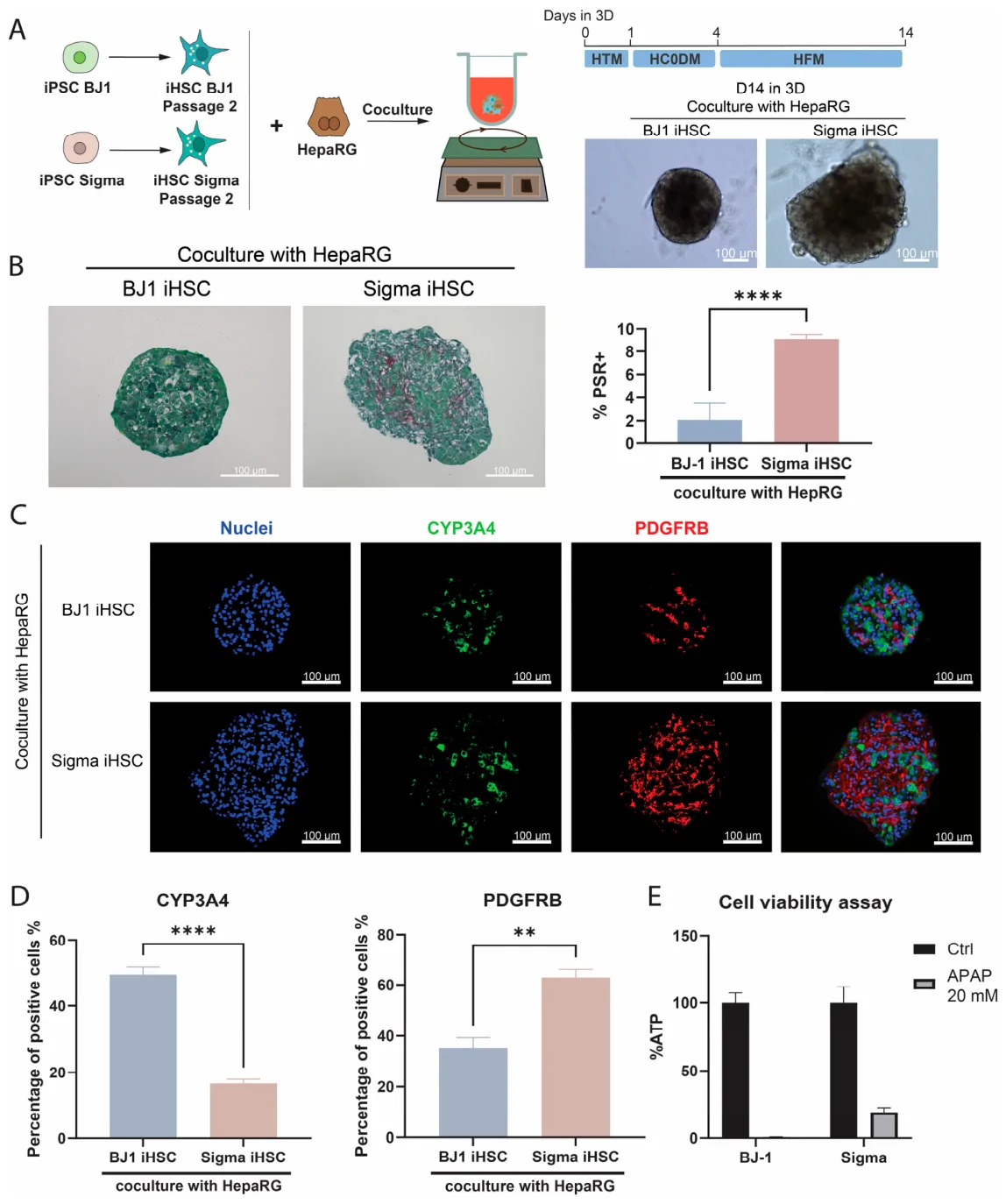
HSCs from different sources influence fibrosis level and hepatic functionality in liver organoids. (**A**) Schematic overview of the liver organoid culture workflow and representative brightfield images of organoids after 14 days of 3D culture. HepaRG cells were co-cultured with BJ1 iHSCs P2 or Sigma-iHSCs P2 in HepaRG culture medium. Scale bar: 100 μm. (**B**) Representative Picrosirius Red/Fast Green staining images of liver organoids generated with BJ1-iHSCs or Sigma-iHSCs, together with quantification of the percentage of Picrosirius Red-positive (PSR+) area. Data are shown as mean ± SEM. **** *p* < 0.0001. *n* = 3–9 organoids per condition. (**C**) Representative immunofluorescence images of liver organoids stained for nuclei, CYP3A4, and PDGFRB. Merged images are shown. Scale bar: 100 μm. (**D**) Quantification of the percentage of CYP3A4-positive and PDGFRB-positive cells normalized to total DAPI-positive nuclei in the organoids shown in (**C**). Data are shown as mean ± SEM. ** *p* < 0.01; **** *p* < 0.0001. *n* = 3–6 organoids per condition. (**E**) Cellular ATP levels in liver organoids after 72 h treatment with 20 mM APAP. *n* = 11–12, with 6 organoids per repeat.

To examine whether BJ1-iHSCs and Sigma-iHSCs behave differently when cultured with hepatocytes, we generated hepatocyte-HSC 3D co-cultures. Following a previously established method, Passage 2 Sigma-iHSCs or BJ1-iHSCs were co-cultured with HepaRG cells in cell repellent plates under orbital shaking conditions to generate matrix-free liver organoids (Figure 2A) [20]. HepaRG cells are derived from a hepatocarcinoma, and when differentiated towards hepatocytes, they exhibit functions comparable to those of primary human hepatocytes (PHH) [31], especially when cultured in 3D [32]. No obvious morphological differences were observed under brightfield microscopy at days 1 and 4 of co-culture. By day 4, BJ1-iHSC/HepaRG organoids did display a more rounded boundary (Figure S1), whereas Sigma-iHSC/HepaRG organoids retained a rougher boundary and appeared larger in size from day 4 to day 14 (Figure 2A, right panel). Given the central role of HSCs in ECM deposition during fibrosis, we next asked whether there was a difference in matrix production. Sirius red staining confirmed marked differences in collagen deposition, with Sigma-iHSC/HepaRG organoids showing a significantly higher percentage of Sirius red-positive area than the other two organoid types, whereas BJ1-iHSC/HepaRG organoids contained only limited amounts of collagen (Figure 2B). This is also consistent with the findings of Kazemzadeh Dastjerd et al. [19], who reported greater collagen accumulation in Sigma-iHSC-based 3D co-cultures when compared with pHSC-containing organoids. Together, these data indicate that Sigma-iHSCs maintain a matrix-producing phenotype when co-cultured with HepaRGs, while BJ1-iHSCs behave more like pHSCs by obtaining a less activated phenotype.

HSCs are known to influence hepatocyte function during liver fibrosis [33], and activated HSCs have been associated with reduced CYP3A4 activity in hepatocyte co-culture systems [34]. To determine whether BJ1- and Sigma-derived iHSCs differentially affect the hepatocyte phenotype, organoids were stained for the mature hepatocyte marker CYP3A4 and the HSC marker PDGFRB (Figure 2C,D). Quantification of CYP3A4-positive cells suggests that the source of HSCs influenced the hepatocyte-associated phenotype of HepaRG cells within the organoids. In parallel, Sigma-iHSC/HepaRG organoids contained the highest proportion of PDGFRB-positive cells, indicating source-dependent differences in HSC marker representation within the 3D organoid context (Figure 2D). To further assess functional differences, organoids were exposed to 20 mM APAP for 72 h. ATP levels decreased almost to zero in BJ1-iHSC/HepaRG organoids, whereas Sigma-iHSC/HepaRG organoids retained lower but detectable ATP levels, indicating that the hepatocytes retain a higher functionality in the BJ1-iHSC/HepaRG than in the Sigma-iHSC/HepaRG organoids (Figure 2E). Overall, these results suggest that HSCs from different sources differentially shape liver organoid phenotypes, affecting organoid morphology, ECM deposition, hepatocyte functionality and injury response.

### 3.3. Single-Cell Transcriptomic Profiling Reveals Divergent States of iHSCs Despite Conserved Cellular Identity

**Figure 3:**
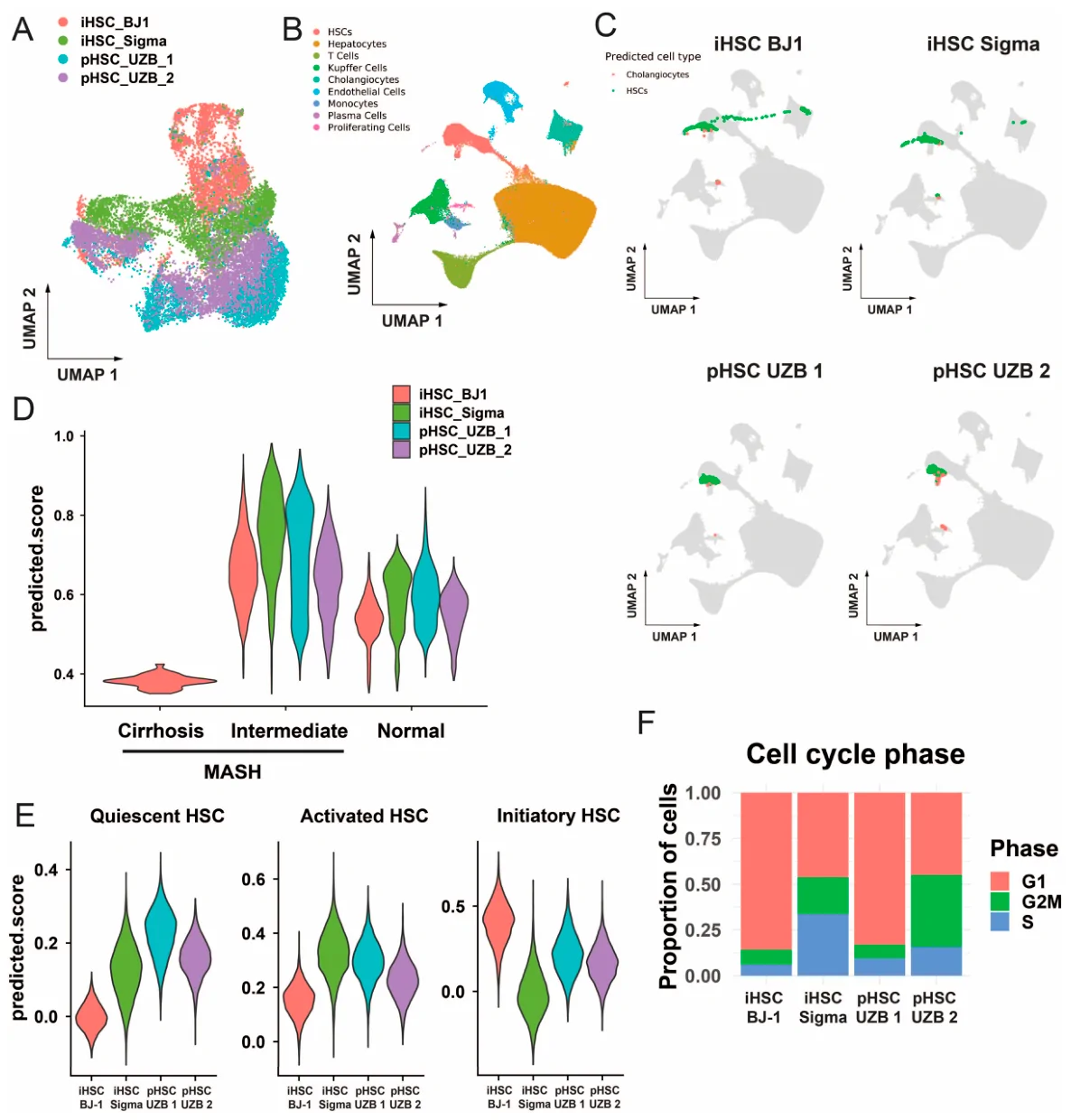
scRNAseq analysis reveals similarities and distinct cellular states among BJ1-iHSCs, Sigma-iHSCs and donor-derived pHSCs cultured in 2D. (**A**) UMAP visualization of BJ1-iHSCs, Sigma-iHSCs, and 2D-cultured pHSCs from two donors in the integrated scRNAseq dataset. (**B**) UMAP visualizations of single-nucleus RNA-seq data from human liver samples reported by Sugimoto et al. [33], including samples from normal, intermediate MASH, and cirrhotic MASH conditions. Cells are labelled by annotated cell type. (**C**) Projection of BJ1-iHSCs, Sigma-iHSCs, and pHSCs onto the Sugimoto et al. reference UMAP (Figure S2A), shown as three separate panels. Reference cells are shown in grey and projected query cells are coloured according to predicted cell type (cholangiocytes or HSCs). (**D**) Violin plot of predicted similarity scores after reference-based mapping of BJ1-iHSCs and Sigma-iHSCs to HSC populations from the normal, intermediate MASH, and cirrhotic MASH conditions in the Sugimoto et al. dataset. (**E**) AUCell scoring of human quiescent HSC, activated HSC, and initiatory HSC gene signatures from Merens et al. [35], shown as violin plots for BJ1-iHSCs, Sigma-iHSCs and pHSCs. (**F**) Cell cycle phase distribution of BJ1-iHSCs, Sigma-iHSCs, and pHSCs, shown as proportions of cells assigned to G1, S, and G2/M phases.

To further define the transcriptional states of BJ1-iHSCs and Sigma-iHSCs, we profiled 2D-cultured cells using single-cell RNA sequencing (scRNAseq) and compared them with donor-derived 2D-cultured pHSCs. Donor characteristics and related information for the primary HSCs are provided in Table 1. UMAP visualization shows that BJ1-iHSCs, Sigma-iHSCs, and pHSCs occupied partially overlapping but distinct regions in the integrated transcriptional space (Figure 3A). To assess cellular identity, we mapped BJ1-iHSCs, Sigma-iHSCs, and pHSCs to the human liver reference dataset from Sugimoto et al. [33] (Figure 3B). Cell type prediction indicated that all three populations were predominantly assigned to HSC populations, with a very small fraction assigned to cholangiocytes (Figure 3C and Figure S2A), indicating successful differentiation of both BJ1- and Sigma-derived cells towards HSCs. Notably, the two pHSC preparations also differed in their mapping profiles, with UZB2 showing a higher proportion of cells assigned to cholangiocyte identity than UZB1, suggesting donor-dependent variability among primary HSCs. In a stage-specific comparison, both iHSC and pHSC populations showed higher predicted similarity scores to HSCs isolated from patients suffering from intermediate MASH than to normal or cirrhotic MASH patients (Figure 3D and Figure S2B), suggesting that both iHSCs and passaged pHSCs resemble an intermediate rather than an advanced disease-associated state. Together, these analyses support the HSC-like identity of both iHSC populations, while also indicating detectable donor-dependent differences among the pHSC preparations.

While iPSC-derived HSCs were generated in regular 2D culture conditions, which also activate primary HSCs [34], their ability to recapitulate primary HSC states remains an important question. To address this, we next evaluated HSC state-associated gene signatures from Merens et al., including quiescent HSCs, fully activated HSCs, and initiatory HSCs, the latter representing HSCs isolated after an acute liver injury [35] (Figure 3E). Compared to pHSCs, BJ1-iHSCs were characterized by a high initiatory HSC signature activity but low quiescent- and activated HSC signature activity, suggesting an initiatory-like state rather than a fully activated phenotype. By contrast, Sigma-iHSCs showed higher activated HSC signature scores than pHSCs, indicating a stronger activated-HSC transcriptional programme. Within the pHSC group, UZB1 and UZB2 also showed donor-dependent differences, with UZB1 displaying higher quiescent- and activated HSC signature scores than UZB2 (Figure 3E). As the two iHSC populations displayed distinct HSC-related transcriptional signatures, we next examined whether they differed in proliferative state. BJ1-iHSCs were predominantly assigned to G1 phase, whereas Sigma-iHSCs contained the highest proportion of S-phase cells. Interestingly, the two pHSC preparations also differed in their cell cycle profiles: UZB1 showed a more G1-dominant distribution, similar to BJ1-iHSCs, whereas UZB2 contained higher proportions of S- and G2/M-phase cells, approaching the overall cycling fraction observed in Sigma-iHSCs. However, both pHSCs contained lower proportions of S-phase cells than Sigma-iHSCs (Figure 3F). Together, these analyses indicate that both BJ1-iHSCs and Sigma-iHSCs have clear HSC identities but differ from 2D-cultured pHSCs; BJ1-iHSCs are more strongly associated with an initiatory-like HSC state, whereas Sigma-iHSCs display more activated and proliferative HSC-associated features. Such differences in HSC states are also observed when comparing two primary HSC cultures.

### 3.4. Differential Pathway Enrichment Highlights Proliferation and ECM Remodelling Divergence Between BJ1 and Sigma iHSCs

**Figure 4:**
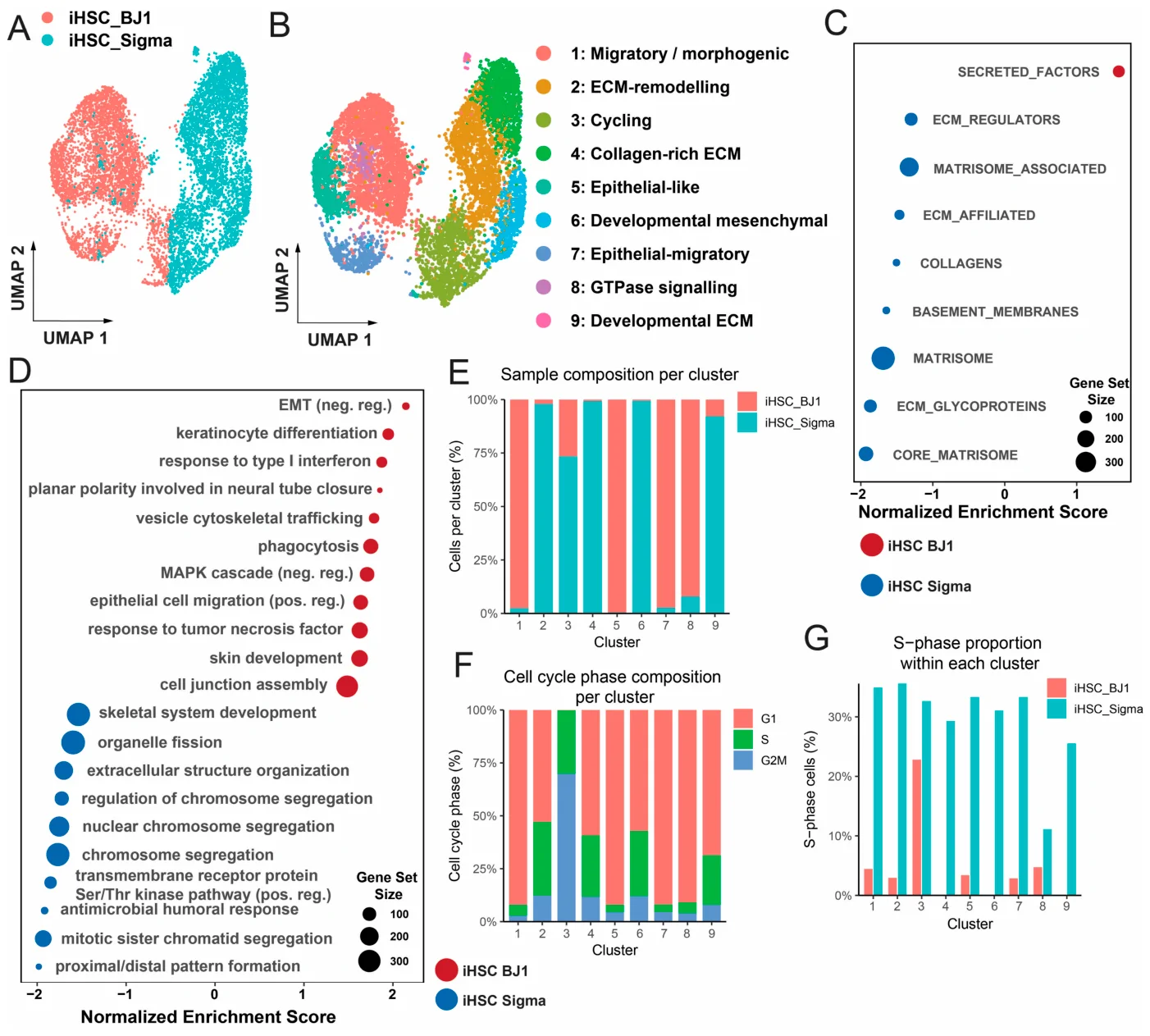
Pathway analyses reveal proliferative, extracellular matrix-related, and metabolic differences between BJ1- and Sigma-derived iHSCs. (**A**) UMAP visualization of BJ1-iHSCs and Sigma-iHSCs cultured in 2D. (**B**) UMAP visualization of annotated iHSC clusters. Cluster annotations were assigned based on top enriched GO biological processes and cluster-specific differentially expressed genes (DEGs). The top five enriched GO pathways for each cluster are shown in Figure S3, and the full list of DEGs is provided in Table S1. (**C**) GSEA of NABA matrisome gene sets in BJ1-iHSCs and Sigma-iHSCs. Most matrisome-associated pathways were positively enriched in Sigma-iHSCs, indicating broad enrichment of extracellular matrix-related programmes. (**D**) GO enrichment analysis of the shared cluster identified in the combined iHSC dataset, showing strong enrichment of proliferation-associated biological processes. (**E**) Proportions of BJ1-iHSCs and Sigma-iHSCs within each cluster identified in the iHSC dataset. (**F**) Distribution of G1, S, and G2/M phase assignments within each iHSC cluster. (**G**) Proportion of S-phase cells within each iHSC cluster, shown separately for BJ1-iHSCs and Sigma-iHSCs.

Given the clear difference in HSC state-associated signatures and cell cycle phase distribution between BJ1-iHSCs and Sigma-iHSCs, we next examined whether broader functional programmes also differed between the two iHSC populations. UMAP visualization showed that the two iHSC populations occupied both overlapping and distinct regions in transcriptional space, indicating that they shared common transcriptional states while also retaining source-dependent differences (Figure 4A). We then identified cluster-specific differentially expressed genes (Table S1) and performed GO enrichment analysis (Figure S3, Table S2), and the top GO per cluster was visualized on the UMAP (Figure 4B). Sigma-enriched clusters were associated with ECM remodelling, collagen-rich matrix production, and developmental/mesenchymal programmes. In contrast, BJ1-enriched clusters were associated with migratory, morphogenesis-related, and small GTPase signalling-associated programmes, while clusters 4 and 6 showed epithelial-like transcriptional features.

Consistently, gene set enrichment analysis (GSEA) of NABA matrisome gene sets [36,37] showed broad positive enrichment of matrisome-associated programmes in Sigma-iHSCs, indicating enhanced extracellular matrix-related transcriptional activity in this population (Figure 4C). GSEA based on GO terms confirmed distinct biological programmes in the two populations (Figure 4D), with BJ1-iHSCs in a comparatively less activated state and Sigma-iHSCs in a more activated state.

We next assessed the cluster composition of the iHSC dataset and its relationship with cell cycle state. Sample composition analysis showed that clusters 1, 5, 7, and 8 were predominantly composed of BJ1-iHSCs, whereas clusters 2, 4, 6, and 9 were mainly contributed by Sigma-iHSCs. Cluster 3 was shared by both iHSC populations (Figure 4E). The shared cluster 3 was annotated as a cycling cluster, consistent with its enrichment for S- and G2/M-phase cells (Figure 4F). Together, these analyses suggest that Sigma-iHSCs preferentially occupy proliferative and ECM-associated transcriptional states, whereas BJ1-iHSCs are enriched for migratory/morphogenesis-associated and epithelial-like states, with both iHSC populations contributing to a shared cycling cluster. Cell cycle phase analysis further showed that the BJ1-enriched clusters were largely composed of G1-phase cells, whereas the Sigma-enriched clusters contained lower proportions of G1-phase cells and higher proportions of cycling cells. Notably, the shared cluster 3 was almost entirely composed of S- and G2/M-phase cells, indicating that it represented a cycling population present in both iHSC lines (Figure 4F). Sigma-iHSCs consistently showed higher S-phase representation than BJ1-iHSCs, suggesting that Sigma-iHSCs are more proliferative (Figure 4G).

Together with the 3D organoid phenotypes described above, where the Sigma-iHSC/HepaRG organoids have a bigger size and show more ECM deposition, these transcriptomic analyses support a model in which Sigma-iHSCs preferentially adopt a proliferative, ECM-remodelling, and activation-associated state, whereas BJ1-iHSCs display a less ECM-producing profile with distinct migratory and morphogenesis-associated transcriptional features.

### 3.5. Heterogeneity in Patient-Derived HSCs Also Contributes to Divergent Fibrotic Responses in 3D Liver Organoids

**Figure 5:**
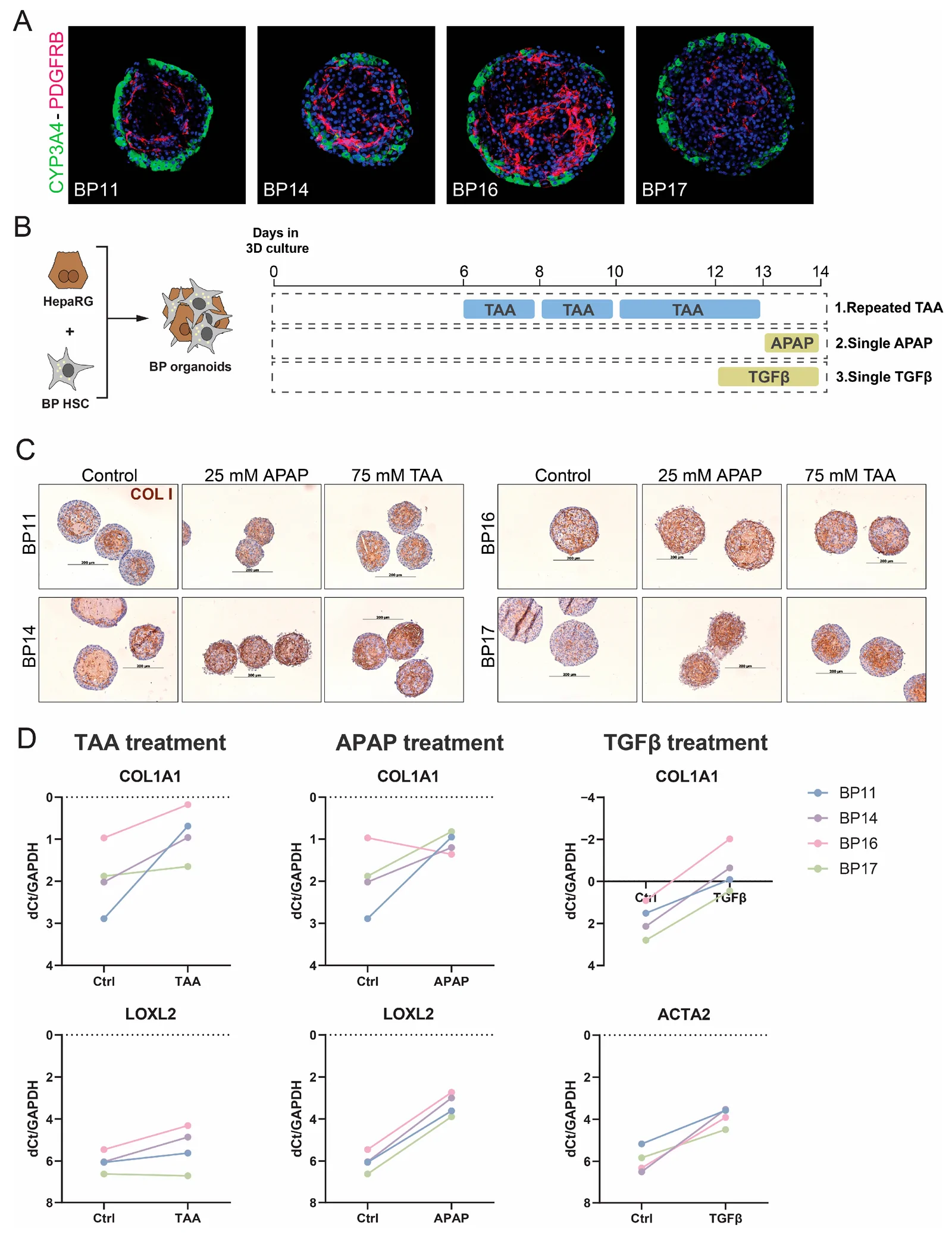
Donor-dependent heterogeneity of primary HSCs contributes to divergent fibrogenic responses in 3D liver organoids. (**A**) Representative immunofluorescence images of liver organoids generated with HepaRG and primary HSCs from different donors, stained for CYP3A4 and PDGFRB. Distinct HSC donors resulted in different PDGFRB-positive areas within the organoids. (**B**) Schematic overview of the treatment regimens used for the organoid experiments, including single APAP treatment, repeated TAA treatment, and 48 h TGFβ treatment. (**C**) Representative immunostaining images of organoids generated with four different pHSC donors under control, 25 mM APAP, or 75 mM TAA treatment conditions. Scale bar: 200 μm. (**D**) qPCR analysis of COL1A1 and LOXL2 in organoids containing different commercial pHSC batches after APAP or TAA treatment, and COL1A1 and ACTA2 after 48 h TGFβ treatment, shown as dCt relative to GAPDH. Lower ΔCt values indicate higher transcript expression. Six organoids were pooled per donor and condition to obtain enough RNA for qPCR analysis.

Since our analyses of iPSC-derived HSCs revealed substantial source-dependent heterogeneity, we next examined whether primary HSC preparations from different donors likewise contribute to divergent responses in 3D liver organoid models. To this end, primary human HSCs from different donors (BP11, 14, 16, and 17, Table 1) were co-cultured with HepaRGs to form organoids. Immunofluorescence staining for CYP3A4 and PDGFRB under standard culture conditions revealed substantial donor-dependent differences in PDGFRB-positive areas, indicating different HSC activation statuses within the cultures (Figure 5A). We evaluated whether these donor-dependent differences persisted under injury- and fibrosis-related stimulation by exposing these spheroids to APAP (25 mM) or thioacetamide (TAA, 75 mM), two commonly used liver injury-associated stimuli that can promote fibrogenic responses in liver models [38] and TGFβ (25 ng/mL), a direct stimulator of HSC activation (Figure 5B). Collagen staining of control (unchallenged) organoids showed marked differences between pHSC donors, which seemed to influence the potential of the HSCs to respond to the injury. BP16, which displayed high PDGFRB and collagen positivity in control organoid conditions, did not really increase in collagen positivity upon TAA or APAP exposure, while BP17 HSCs had low PDGFRB/Collagen expression and showed a clear response to both TAA and APAP (Figure 5C). This qualitative difference in activation was further supported at the transcriptional level by qPCR analysis of COL1A1 and LOXL2 (Figure 5D), which revealed variability in both the basal transcriptional state and treatment-associated response patterns of pHSC-containing organoids. In control conditions, COL1A1 expression differed between individual BP-derived organoid preparations, with differences of approximately 1–2 dCt values in some cases, indicating that the starting activation state was not uniform across samples. In addition, selected donor–treatment combinations showed distinct response patterns: COL1A1 expression decreased after APAP treatment in BP16, while BP11, 14 and 17 showed increased COL1A1 expression following the same APAP treatment. In addition, LOXL2 and COL1A1 expression remained largely unchanged in BP17 after TAA exposure, while BP11, 14 and 16 showed a response to TAA exposure. Taken together, these findings suggest that human liver donor variability is retained in primary HSC-containing organoids and may contribute to differences in both baseline and stimulus-induced fibrogenic phenotypes in 3D co-culture.

## 4. Discussion

Our study suggests that source-dependent differences in HSCs can substantially influence fibrotic and functional phenotypes in human liver organoid models. We found that iHSCs generated from two different iPSC lines exhibited distinct phenotypes during 2D differentiation, and that these differences displayed divergent outcomes in organoid cultures with HepaRG cells. scRNAseq analyses showed that these iHSC populations occupied distinct transcriptional states associated with differences in proliferative activity, extracellular matrix-related programmes, and activation-associated features. Importantly, pHSCs from different donors also showed clear transcriptional profile differences and divergent responses to hepatocyte injury. Together, these findings suggest that the human HSC source is not a trivial technical variable, but an important contributor to hepatic organoid behaviour.

A key implication of this work is that variation between iPSC sources or primary human HSCs should be viewed not only as evidence of protocol reproducibility, but also as a source of biologically meaningful variability that can influence organoid phenotypes. Many studies have successfully differentiated iPSCs to iHSCs or improved the differentiation protocols [10,11,15,17,19,39,40,41]; however, few studies have directly compared iHSCs derived from different iPSC sources. The importance of cell source in multicellular liver organoid systems has been recognized previously by Nie et al. [42] who generated liver organoids using single donor-derived hiPSC endoderm, endothelial cells, and mesenchymal cells, highlighting the relevance of donor-consistent cellular components in organoid construction [43]. More broadly, iPSC lines should not be considered interchangeable starting materials, as donor genetic background, tissue of origin, epigenetic memory, and reprogramming or culture history can influence differentiation outcomes and cellular state [44,45,46,47]. Our transcriptional analyses support the interpretation that iPSC source can influence the state of the resulting iHSCs. Rather than appearing as transcriptionally uniform populations, BJ1-iHSCs and Sigma-iHSCs contained distinct subpopulations with differences in proliferative, ECM-remodelling, mesenchymal/developmental, migratory, and epithelial-like programmes. More specifically, BJ1-iHSCs were enriched for programmes related to negative regulation of MAPK signalling, inflammatory responsiveness, epithelial differentiation, and cell junction organization, consistent with a comparatively less matrix-producing and more cell-organization-associated profile. In contrast, Sigma-iHSCs were enriched for proliferation-associated processes, extracellular structure organization, and receptor serine/threonine kinase signalling, including pathways related to TGF-β signalling [48,49], supporting a more proliferative, ECM-remodelling, and activation-associated state. The enrichment of Sigma-iHSCs in S and G2/M phases also supports this interpretation, as activated HSCs are generally associated with increased proliferative activity [50,51]. These transcriptional states were functionally validated in our 3D co-culture system, where Sigma-iHSC/HepaRG organoids deposited significantly higher amounts of collagen and retained a larger, more proliferative structure. Conversely, BJ1-iHSCs displayed a less active, initiatory-like transcriptional state, which functionally translated into minimal baseline matrix deposition in 3D. Such characterization could be particularly important for building more physiologically representative liver organoids, where HSC state [35,52], zonation-associated functions [33,53], and cell–cell interactions [54,55] may influence organoid maturation and disease-modelling capacity. Additionally, BJ1-derived cells showed stronger collagen staining at day 10, whereas Sigma-iHSCs P2 showed stronger ECM-associated transcriptional programmes and Sigma-iHSC/HepaRG organoids displayed stronger matrix-associated readouts in 3D culture. The phenotypic changes between day 10 and passaged iHSCs have been reported and could be at the basis of the discrepancy [17,18]. This further supports the need for stage- and context-specific characterization of the HSC component in liver organoid models.

We show that two iPSC-derived HSC and four primary HSC populations displayed different phenotypes when co-cultured as organoids with HepaRGs. This is in line with previous work showing that cell source and composition can shape liver organoid development, maturation, and function [56,57,58]. Our study extends these observations by focusing specifically on the variability within the HSC component, showing that pHSC donor differences can alter matrix-associated and fibrogenic readouts in 3D co-culture. Although pHSC batch or source is often treated as a secondary methodological detail in liver fibrosis models [12,13,14], our results suggest that variability within the HSC compartment should be considered when designing, comparing, and interpreting fibrosis-related organoid assays. In tumour organoid studies, intra-model cellular and molecular heterogeneity has already been recognized as an important factor affecting drug-response assessment [59,60]. Patient-derived cancer organoids can maintain heterogeneity from the original tissue and support high-throughput drug screening [61]. From a practical perspective, one could, for instance, consider using several different donor-derived iHSCs and/or pHSCs, with a different basal response to a hepatic injury, to represent the heterogeneity present in vivo when modelling liver fibrosis in vitro. For standardized drug-screening reporting, the cellular source, passage number, disease background and baseline fibrosis levels would need to be part of the information accompanying the results of such an in vitro model. Preserving and characterizing donor-dependent HSC heterogeneity may be valuable for future precision-medicine applications, where patient-specific stromal responses could influence therapeutic sensitivity and disease-modelling outcomes.

It is tempting to speculate that genetic imprinting or single nucleotide polymorphisms (SNPs) could be at the basis of these differences. For iPSC-derived hepatocytes, this was elegantly done by deriving 24 iPSC lines and showing a genotype-to-phenotype association for lipid accumulation in iPSC-derived liver organoids based on known SNPs [58]. However, to establish a statistically robust novel causal genotype-to-phenotype relationship for the iHSC or pHSC behaviour in the organoids would be virtually impossible. A recent meta-analysis identified 738 recurrent genetic abnormalities in embryonic stem cells and iPSCs [62]. Besides chromosomal aneuploidy and sub-chromosomal copy number variations, iPSCs harbour on average 10 point mutations in protein-coding regions [63], and clonal and subclonal UV-damage mutations are associated with different chromatin states [64]. Similarly, for primary HSCs, genetic and disease evolution can greatly impact the genetic baseline of HSCs when isolated. Mutations leading to survival advantage in a toxic or fibrotic environment result in a unique mutational landscape per liver or even different regions of the liver [65]. Underlying liver pathology, tumour-associated changes, medication and age therefore may also contribute to donor-dependent HSC variability [66,67,68]. Moreover, a recent study showed that in only 13–17 cell divisions, primary fibroblasts differed in allele frequencies of 58 somatic mutations between pre- and post-cell culture [69]. The pHSCs used in the current study are all isolated from liver metastasis resection material (a highly stressful environment) and have been passaged 4–5 times (perhaps accumulating mutations). Attempting to link their behaviour in the organoids to the donor’s germline or culture-acquired genotype would require a large number of patient-derived iPSCs and donor-HSCs.

Some of our phenotypic interpretations should be considered with caution. In particular, the higher proportion of Sigma iHSCs in S and G2/M phases supports a more proliferative state [70], but should not be taken as a direct or exclusive marker of HSC activation, as cell cycle redistribution may also depend on contextual cues [50]. Likewise, the enrichment of EMT-related pathways and epithelial-like transcriptional features in BJ1 iHSCs should be interpreted carefully. These signatures may not necessarily indicate epithelial identity or direct EMT conversion. Because iHSC differentiation involves intermediate mesodermal and mesenchymal-like states [18,71], epithelial-like signatures may also reflect transient or incompletely resolved differentiation states rather than bona fide epithelial identity. Additionally, the contribution of EMT and epithelial-lineage plasticity to liver fibrosis remains controversial [72,73], and additional marker validation will be required to determine the precise identity of these epithelial-like clusters. These features are therefore better viewed as supportive components of a broader phenotypic difference rather than as definitive standalone indicators.

More generally, our conclusions are based on in vitro differentiation and organoid systems, and the source-dependent states observed here may not fully recapitulate the full spectrum of HSC diversity and response in vivo. For instance, the treatments used to model liver fibrosis in this study do not represent the full spectrum of liver injury and fibrosis mechanisms. However, a recent HSC single-cell atlas reported a highly similar HSC activation process across different liver disease aetiologies [35]. Even though this atlas primarily focused on mouse liver injury models, the integrated human HSC datasets, including HSCs from healthy livers and from patients with Crigler–Najjar, obesity and cirrhosis, showed a similar HSC activation trajectory. This supports the concept that shared HSC activation programmes may operate across distinct injury contexts and species. It suggests that the donor-dependent differences observed here may be relevant beyond the specific injury conditions tested in this study, although additional injury contexts will be required to define the generality of these findings. In addition, the primary HSC analyses were constrained by the number of available batches and sources, and broader benchmarking across additional donors and cell preparations will be needed to define the generality of these effects.

In conclusion, our findings indicate that source-dependent HSC variability is an important biological factor of fibrotic-associated readouts and response heterogeneity in human liver organoid models. More systematic characterization and standardization of the HSC component should therefore improve the robustness, interpretability, and translational value of multicellular liver fibrosis models.

## Figures and Tables

**Table 1:** Patient characteristics of primary HSCs. Overview of human patient characteristics of the donor livers used for the isolation of primary HSCs. CRC: colorectal cancer, NA: not available. Cell viability was determined with trypan blue.

HSC	Age	Gender	Liver Disease	Medication	#Cells	Viability	Application
UZB1	55	Male	Liver metastasis (CRC)	NA	7,850,000	NA	scRNAseq
UZB2	59	Male	Liver metastasis (CRC)	NA	2,950,000	NA	scRNAseq
BP11	60	Female	Cholangiocarcinoma and liver metastasis (CRC)	Arestal, Alprazolam	330,000	93%	HepaRG coculture
BP14	59	Female	Liver metastasis	Chemotherapy: Crestor, Lexomil, Stilnox, Doliprane, Forlax	370,000	92%	HepaRG coculture
BP16	77	Male	Liver metastasis (CRC)	Chemotherapy: NA	670,000	91%	HepaRG coculture
BP17	54	Female	Liver metastasis	Chemotherapy: NA	540,000	80%	HepaRG coculture

**Table 2:** Primer list.

Target Gene Name	Primer Sequence
COL1A1	TCCTGCATCCCCCATAGTTA (For) CTTCAGGAACAGCCACCAGT (Rev)
ACTA2	CTGTTCCAGCCATCCTTCAT (For) TCATGATGCTGTTGTAGGTGGT (Rev)
LOXL2	GGAGAGGACATACAATACCAAAGTG (For) CCATGGAGAATGGCCAGTAG (Rev)
GAPDH	AGCCACATCGCTCAGACAC (For) GCCCAATACGACCAAATCC (Rev)

**Table 3:** Antibodies used for immunofluorescence staining and immunohistochemistry.

Antibody	Source	Cat#
COL1A1 (1/100)	SouthernBiotech	1310-01
PDGFRB (1/100)	Abcam	ab32570
DAPI (1/1000)	Merck	D9564
CYP3A4 (1/200)	Cypex limited UK	MA5-17064
Alexa fluor 488	Thermo Fisher Scientific	A11055
Alexa fluor 594	Thermo Fisher Scientific	A21207

## Data Availability

RNAseq and other data generated in this study have been deposited at Zenodo and are publicly available as of the date of publication (https://doi.org/10.5281/zenodo.20595976). Any additional information required to reanalyze the data reported in this paper is available from the corresponding author upon request.

## Supplementary Materials

The following supporting information can be downloaded at: https://www.mdpi.com/article/10.3390/cells15171556/s1, Supplementary Scheme S1: A schematic overview of the experimental design, including the main cell sources, model systems and experimental readouts used in this study; Figure S1: Early morphological differences among liver organoids generated with different HSC populations; Figure S2: Reference datasets used for assessing the cellular identity of 2D-cultured iHSCs; Figure S3: GO enrichment analysis of cluster-specific differentially expressed genes (DEGs) in BJ1- and Sigma-derived iHSCs; Table S1: DEGs of each cluster in the BJ1-iHSC and Sigma-iHSC combined dataset; Table S2: Enriched GO pathways of each cluster in the BJ1-iHSC and Sigma-iHSC combined dataset.

## Abbreviations

The following abbreviations are used in this manuscript:

HSC	Hepatic stellate cell
iPSC	Induced pluripotent stem cell
iHSC	Induced pluripotent stem cell-derived hepatic stellate cell
pHSC	Primary human hepatic stellate cell
ECM	Extracellular matrix
LDM	Liver differentiation medium
APAP	Acetaminophen (Paracetamol)
TAA	thioacetamide
ANOVA	Analysis of variance
DMSO	Dimethyl sulfoxide
GSEA	Gene set enrichment analysis
EMT	Epithelial–mesenchymal transition
BMP4	Bone morphogenetic protein 4
aFGF	Acidic fibroblast growth factor
HGF	Hepatocyte growth factor
EGF	Epidermal growth factor
