# Supplementary material for: Behaviour of iPSC-Derived and Donor-Derived Hepatic Stellate Cells in Organoid Cultures Is Highly Variable and Contributes to Fibrotic Outcome
Source: Cells. 2026 Aug 28;15(17):1556. doi: 10.3390/cells15171556 (PMC13565511; doi:10.3390/cells15171556)
Supplement: Supplementary file 1 [file cells-15-01556-s001.zip › Yang et al_Suppplementary schemes and figures.pdf]

Behaviour of iPSC-derived and donor-derived hepatic stellate cells in organoid cultures is highly variable and contributes to fibrotic outcome.

Yuwei Yang [1], Mina Kazemzadeh Dastjerd [1, 2], Ayla Smout [1], Vincent Merens [1, 5], Burak Toprakhisar [1, 3], Catherine M. Verfaillie [3], Christophe Chesné [4] and Leo A. van Grunsven [1, *]

[1] Liver Cell Biology Research Group, Vrije Universiteit Brussel, 1090 Brussel, Belgium; yuwei.yang@vub.be
[2] Current address: Department of Micro- and Nanosystems (MST), SciLifeLab, KTH Royal Institute of Technology, Stockholm, Sweden; minakaz@kth.se
[3] Stem Cell Institute Leuven, Katholieke Universiteit Leuven, Herestraat 49, 3000 Leuven, Belgium
[4] Biopredic International SARL, Parc de la Bretech, 35760 Saint Grégoire, France; c.chesne@wepredic.com
[5] Current address: Department of Pathology, Oslo university hospital, Oslo, Norway; vincent.merens@medisin.uio.no
* Correspondence: leo.van.grunsven@vub.be

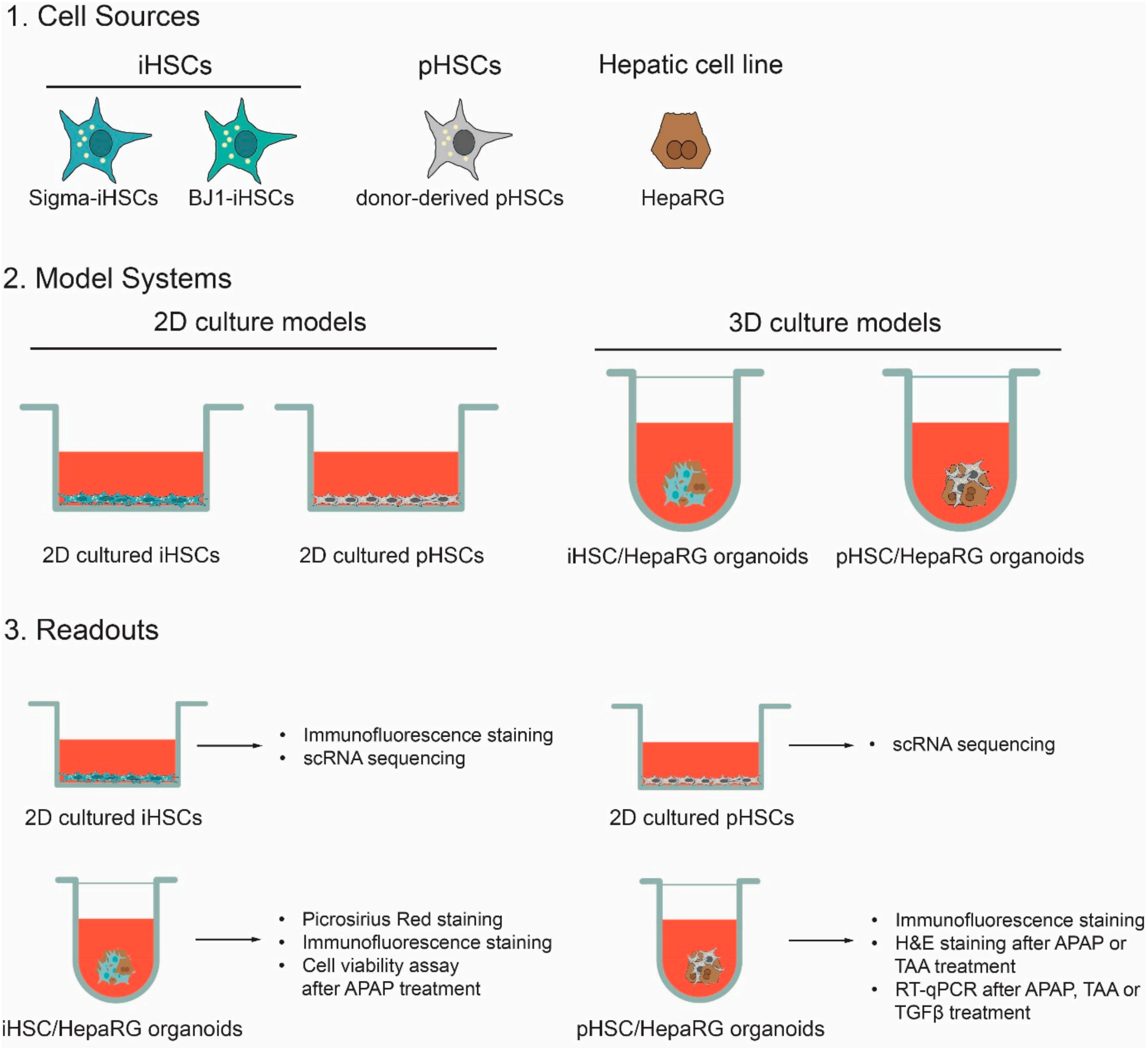

Supplementary Scheme S1: A schematic overview of the experimental design, including the main cell sources, model systems and experimental readouts used in this study.

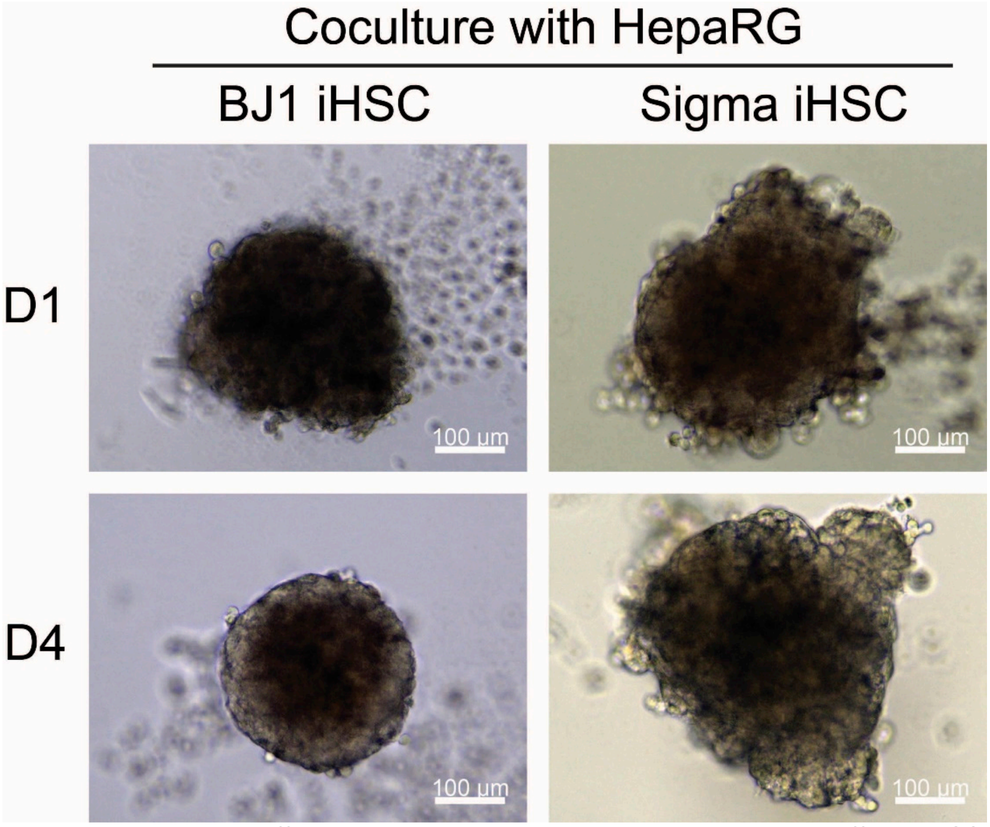

Figure S1. Early morphological differences among liver organoids generated with different HSC populations. Representative brightfield images of liver organoids generated by co-culturing HepaRG cells with BJ1-iHSCs, Sigma-iHSCs, or pHSCs at day 1 and day 4 of 3D culture. BJ1-iHSC/HepaRG and pHSC/HepaRG organoids displayed a more rounded boundary by day 4, whereas Sigma-iHSC/HepaRG organoids retained a rougher boundary and appeared larger in size. Scale bar: 100 µm.

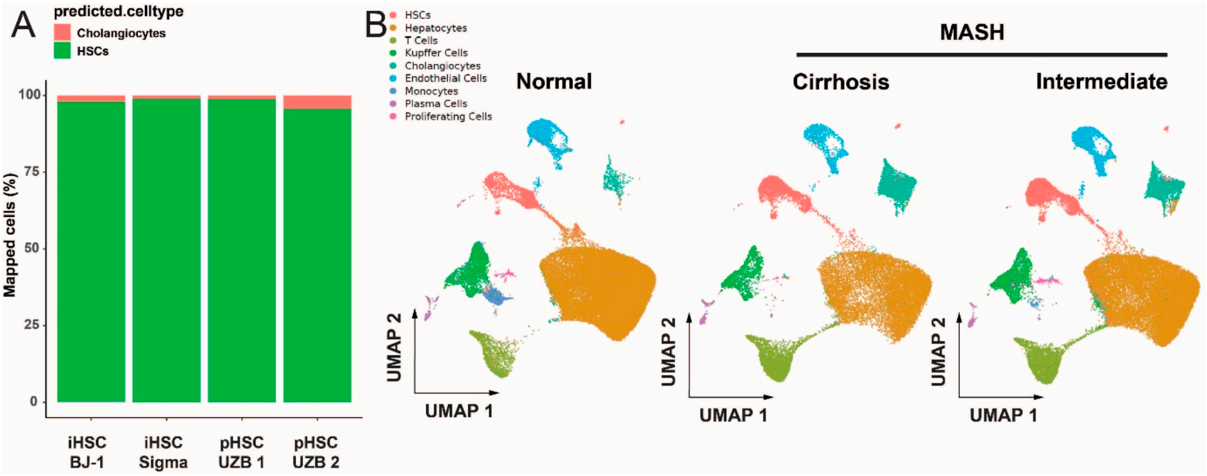

Figure S2. Reference datasets used for assessing the cellular identity of 2D-cultured iHSCs. (A) Stacked bar plot showing the proportions of predicted cell types after mapping BJ1-iHSCs, Sigma-iHSCs, and pHSCs to the human whole-liver reference dataset from Sugimoto et al. [1]. Only cholangiocytes and HSCs identities were predicted in this comparison. (B) UMAP visualizations of the Sugimoto et al. liver reference dataset shown separately for normal, intermediate MASH, and cirrhotic MASH conditions.

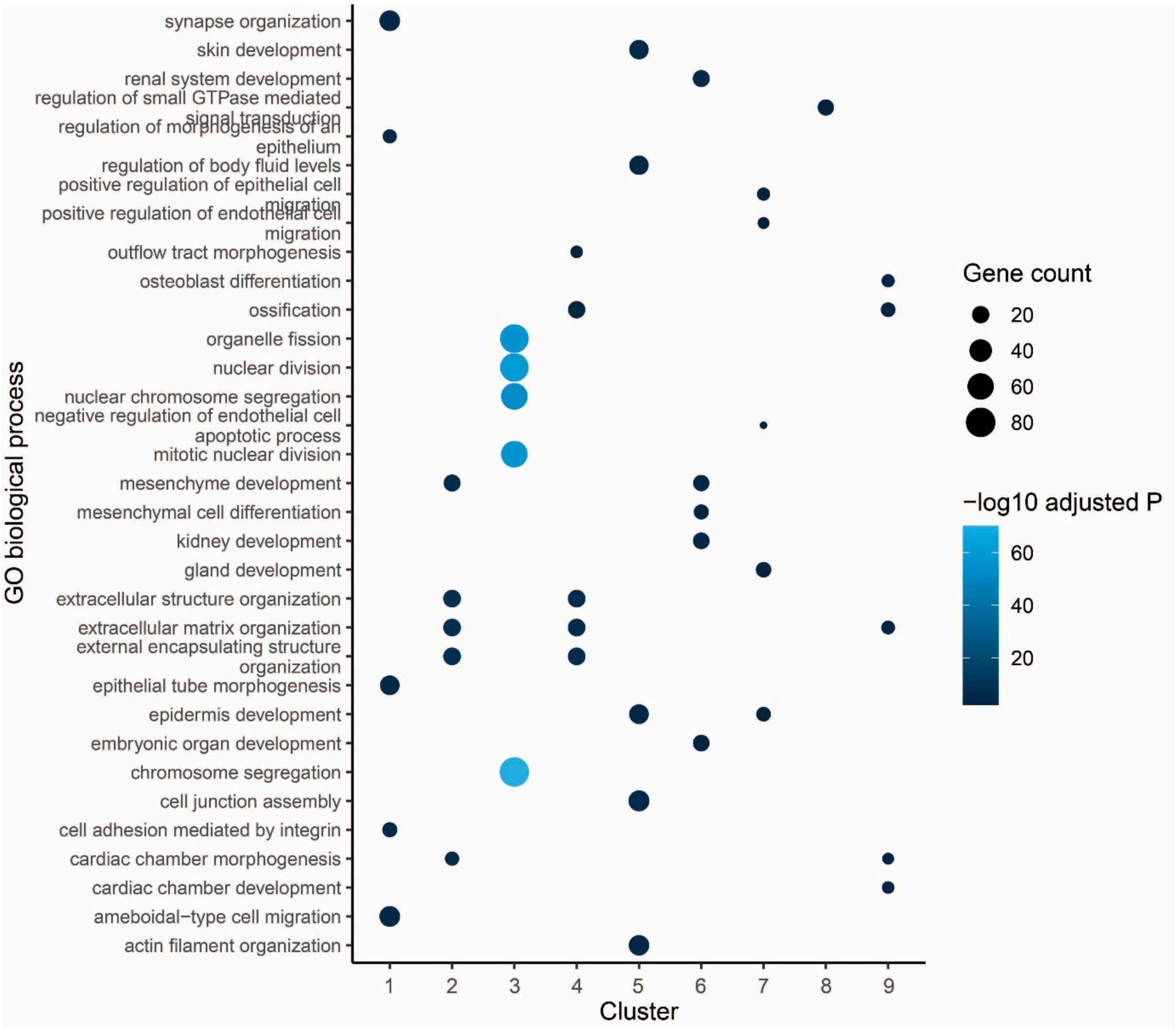

Figure S3. GO enrichment analysis of cluster-specific differentially expressed genes (DEGs) in BJ1- and Sigma-derived iHSCs. Bubble plot showing the top 5 enriched GO biological process terms for each cluster identified in the iHSC dataset.

Reference:

1. Sugimoto, A.; Saito, Y.; Wang, G.; Sun, Q.; Yin, C.; Lee, K.H.; Geng, Y.; Rajbhandari, P.; Hernandez, C.; Steffani, M.; et al. Hepatic stellate cells control liver zonation, size and functions via R-spondin 3. *Nature* **2025**, *640*, 752–761, doi:10.1038/s41586-025-08677-w.
